# The Current Process for the Recycling of Spent Lithium Ion Batteries

**DOI:** 10.3389/fchem.2020.578044

**Published:** 2020-12-03

**Authors:** Li-Feng Zhou, Dongrun Yang, Tao Du, He Gong, Wen-Bin Luo

**Affiliations:** ^1^Section of Environmental Protection (SEP) Key Laboratory of Eco-Industry, School of Metallurgy, Northeastern University, Shenyang, China; ^2^School of Metallurgy, Institute for Energy Electrochemistry and Urban Mines Metallurgy, Northeastern University, Shenyang, China

**Keywords:** spent lithium ion batteries, cathode materials, pyrometallurgical process, hydrometallurgical process, direct physical recycling process

## Abstract

With the development of electric vehicles involving lithium ion batteries as energy storage devices, the demand for lithium ion batteries in the whole industry is increasing, which is bound to lead to a large number of lithium ion batteries in the problem of waste, recycling and reuse. If not handled properly, it will certainly have a negative impact on the environment and resources. Current commercial lithium ion batteries mainly contain transition metal oxides or phosphates, aluminum, copper, graphite, organic electrolytes containing harmful lithium salts, and other chemicals. Therefore, the recycling and reuse of spent lithium ion batteries has been paid more and more attention by many researchers. However, due to the high energy density, high safety and low price of lithium ion batteries have great differences and diversity, the recycling of waste lithium ion batteries has great difficulties. This paper reviews the latest development of the recovery technology of waste lithium ion batteries, including the development of recovery process and products. In addition, the challenges and future economic and application prospects are described.

## Introduction

**Figure 1:**
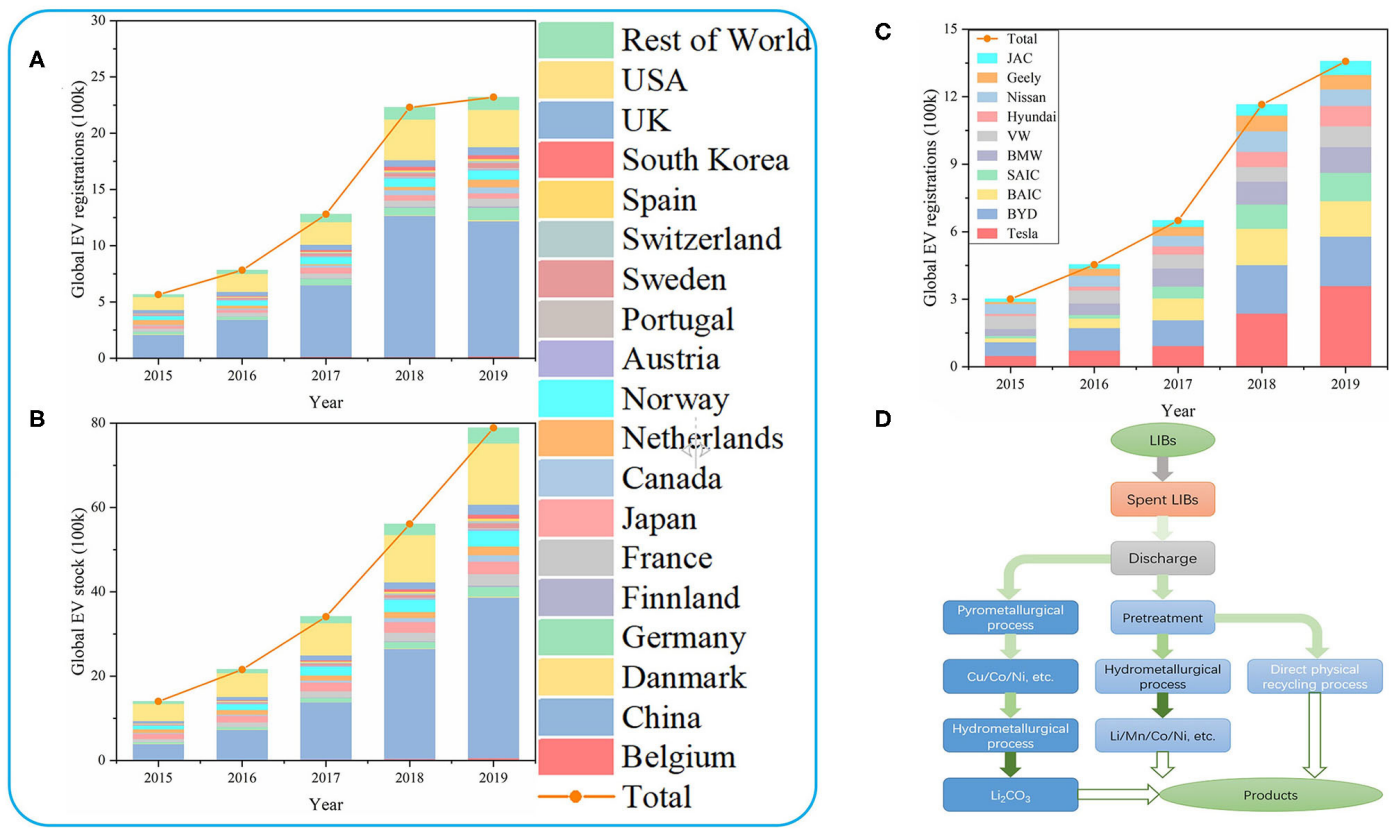
**(A)** The global registration of EVs. **(B)** The global stock of EVs. **(C)** The global company registration of EVs. **(D)** Recovery methods for spent LIBs.

In the early 1990s, Moli and Sony used carbon materials with graphite structure to replace metal lithium anodes, and lithium and transition metal composite oxide such as LiCoO_2_ served as the cathodes, leading to the commercialization of LIBs (Arora et al., 1998; Song et al., 1999; Lee and Lee, 2000; Pattipati et al., 2014). With the popularity of portable electronic devices such as mobile phones and tablet computers in recent years, LIBs have gradually been recognized. What really shakes the lithium resource reserve to promote the development of LIBs is the development of EVs. In the past few years, with the development of energy storage industry, LIBs with higher energy density and higher power output have been widely used in EVs. In particular, attention should be paid to the battery development of BYD and Tesla. At the same time, with the policy orientation and the convenience and benefits brought by EVs, the global EV market is developing rapidly. Figures 1A,B show the development of EVs worldwide, with particular attention to the development of EVs in China and the United States. It can be seen that since 2015, the global stock and registration of EVs have grown rapidly, and China and the United States have shown an amazing growth trend. In 2019, China's EV stock reached 3,810,000, nearly half of the global total and 2.6 times that of the US, and the number of China's EV registrations was 1,204,000, more than half of the global total and almost 4 times that of the US. BYD and Tesla are the two most prominent EV companies, with more than 40% of the world's 1,357,000 registrations in 2019^1^ (Figure 1C). They are represented by LiFePO_4_ and LiNi_1−x−*y*_Co_*x*_Mn_*y*_O_2_ (NCM) (Zhu, 2014). Considering the blowout development of EVs, the disposal of the huge amount of spent lithium-ion batteries (LIBs) brought about by the exhaustion of the life cycle of LIBs will soon become a huge problem that plagues society. Therefore, we should be alert to the upcoming troubles and actively develop the technology and technical level related to the disposal of spent LIBs (Villen-Guzman et al., 2019).

Due to the short history of LIBs, a systematic recycling system has not yet been formed. Generally, a LIB includes a cathode, an anode, a separator, electrolyte, and a case with a sealing function. There are many types of commercial LIBs, mainly including lithium oxides and phosphides, such as LiCoO_2_, LiMn_2_O_4_, LiFePO_4_, NCM, etc. Since 2012, due to the development of EV markets, LiFePO_4_ and LiNi_0.33_Co_0.33_Mn_0.33_O_2_ have occupied more than half of the cathode material market. The electrolyte in the LIBs also contains harmful substances, such as organic solvents and fluorine-containing lithium salts, which may cause great harm to the environment. Therefore, if spent LIBs directly puts in the environment, it will cause an irreversible environmental disaster. In addition, the high-priced metals contained in LIBs, such as Li, Co, Ni, Cu, Al, etc., also have good resource value. In the spent electrode materials, the average content of Li is about 5 wt%, which is much higher than the content in natural ores and has recycling value. Besides, environmental and economic issues are also prerequisites for LIBs recycling. Many organic electrolytes and heavy metal ions have been released into groundwater by battery abandonment and leakage and have proved to be harmful to human health and the ecological environment. In addition, Choubey et al. (2016) reported the economic value of lithium-ion battery recycling, which can generate an economic benefit of $22,000 per ton by calculating the value of lithium and cobalt.

In view of the above reasons, the recycling of spent LIBs has a considerable prospect and is environmentally friendly, so it is imperative to establish a reasonable recycling system and develop related recycling technologies. This article will summarize hydrometallurgical process, pyrometallurgical process and direct physical recycling process (Figure 1D) and summarize the strengths and weaknesses of them (Table 1).

**Table 1:** Comparisons of advantages and disadvantages and challenges for different methods.

**Process**	**Advantages**	**Disadvantages**	**Challenge**
Hydrometallurgical process	High recovery rate High purity product Low energy consumption Less waste gas High Selectivity	More wastewater Long process	Wastewater treatment Optimize the process
Pyrometallurgical process	Simple operation and short flow No requirement for categories and the size of inputs High efficiency	Li and Mn are not recovered High energy consumption Low recovery efficiency More waste gas and the cost of waste gas treatment	Reduce energy consumption and pollution emissions Reduce environmental hazards Combine hydrometallurgy well
Direct physical recycling process	Short recovery route Low energy consumption Environmental friendly High recovery rate	High operational and equipment requirements Incomplete recovery	Reduce recovery costs Lower the requirements for categories Further optimize product performance

## The Methods of Recovering Lithium Ion Batteries

Recycling for LIBs usually involves both physical and chemical processes (Harper et al., 2019). Due to the complex assembly process of LIBs and the wide variety of electrodes, it brings great danger for the recovery of battery. The explosion, combustion and poisonous gas brought on the recovery process are easy to cause casualties. To reduce this risk, spent LIBs usually need to be discharged before recycled. Physical processes usually include pretreatment and direct recovery of electrode materials. These processes usually include disassembly, crushing, screening, magnetic separation, washing, heating treatment, etc. Chemical processes can be divided into pyrometallurgical and hydrometallurgical processes, which usually involve leaching, separation, extraction and chemical/electrochemical precipitation.

### Hydrometallurgical Process

At present, hydrometallurgy is typically used to recover LIBs after pretreatment. According to the physical properties of the materials in the spent LIBs, including morphology, density, and magnetism, etc. The treated battery cases, electrodes and membranes containing electrolytes will be treated separately to improve the safety and recovery rate of hydrometallurgical processes and reduce energy consumption during the use of hydrometallurgical or pyrometallurgical recovery electrode materials. Hydrometallurgy usually involves leaching and reduction. It is usually divided into acid leaching and biological leaching according to the leaching method.

For the acid leaching process, Zhang et al. (1998) reported a recovery rate of over 99% for Co and Li, and Nan et al. (2005) reported a recovery rate of over 98% for Cu. The recovery rate of hydrometallurgy is far from that of pyrometallurgy. In the acid leaching of electrode materials, inorganic acids are mostly used, usually including hydrochloric acid (HCl) (Li et al., 2009; Guo et al., 2016; Barik et al., 2017), sulfuric acid (H_2_SO_4_) (Dun-Fang et al., 2009; Meshram et al., 2015, 2016), nitric acid (HNO_3_) (Li et al., 2011; Mossini et al., 2015), and phosphoric acid (H_3_PO_4_) (Pinna et al., 2016; Chen et al., 2017), while the organic acids studied include citric acid (Li et al., 2010; Gao et al., 2019; Yu et al., 2019), oxalic acid (Zeng et al., 2015), and tartaric acid (Chen et al., 2019).

Lee and Rhee (2003) used HCl to recycle LiCoO_2_ and revealed the principle of acid leaching:

$$\begin{array}{l} 2\text{LiCo}{\text{O}}_{2}+8\text{HCl}\to 2\text{CoC}{\text{l}}_{2}+\text{C}{\text{l}}_{2}+2\text{LiCl}+\;4{\text{H}}_{2}\text{O}. \end{array}$$

**Figure 2:**
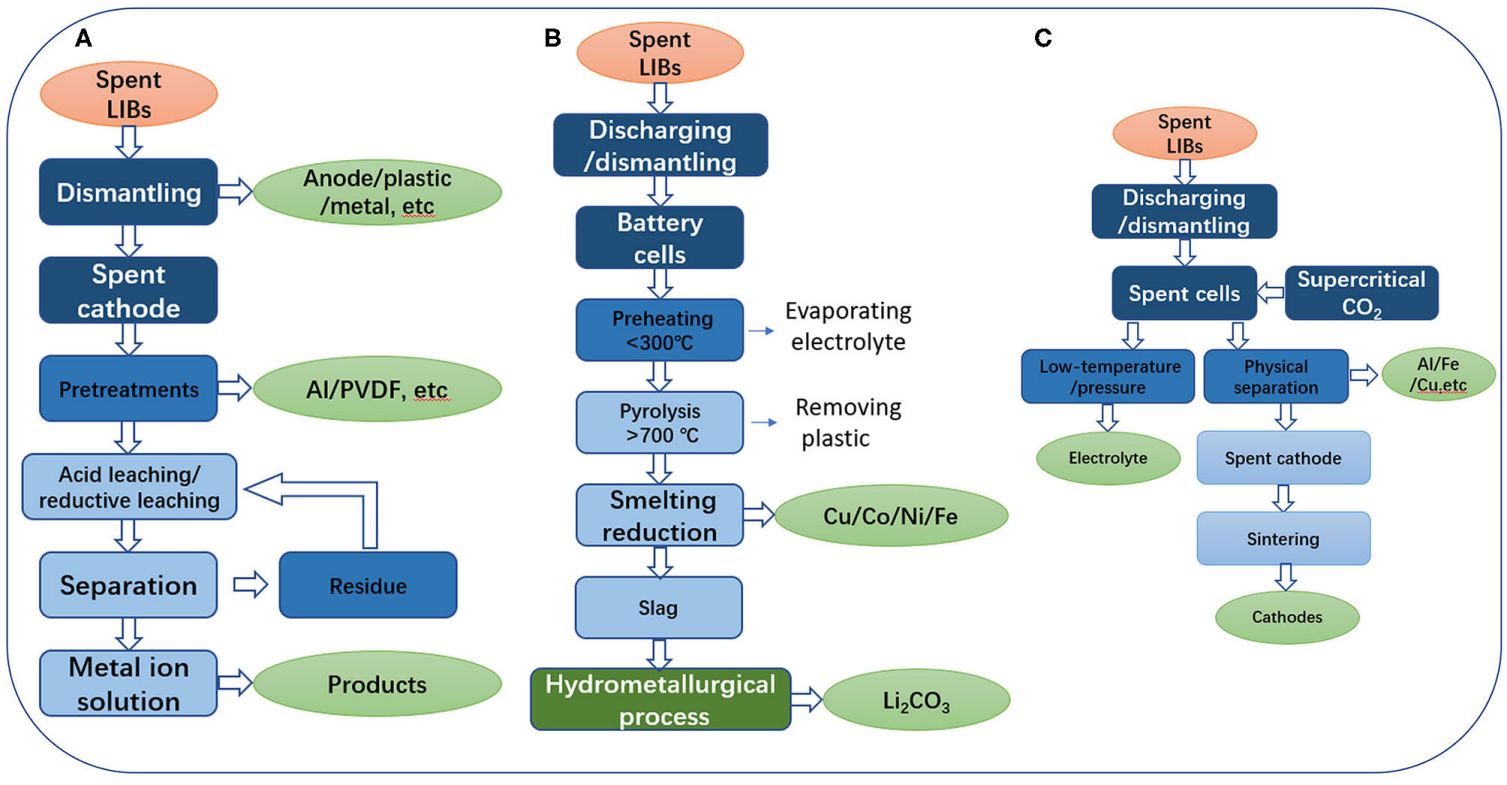
**(A)** Hydrometallurgical process. **(B)** Pyrometallurgical process. **(C)** Direct physical recycling process.

In the process of HCl leaching (Figure 2A), the solution has a strong ability to leach Co from spent LIBs and reduce Co^3+^ to Co^2+^. But the Cl_2_ produced during leaching is a difficult problem to solve because of its toxicity and corrosiveness. For NCM electrode materials, HCl solution with strong acidity should not be used in order to slow the dissolution of Mn. Therefore, Castillo et al. (2002) reported the acid hydrolysis process of cathode materials with nitric acid, and achieved the recovery of 100% Li and 95% Mn. In practical application, one of the best ways to solve the problem is to use H_2_SO_4_ hydrolysis and use hydrogen peroxide (H_2_O_2_) to reduce the Co^3+^ to Co^2+^. The reaction equation is as follows:

$$\begin{array}{l} 2\text{LiCo}{\text{O}}_{2}+3{\text{H}}_{2}\text{S}{\text{O}}_{4}+{\text{H}}_{2}{\text{O}}_{2}\to 2\text{CoS}{\text{O}}_{4}+{\text{O}}_{2}+\text{Li}2\text{S}{\text{O}}_{4}\\ \;+\;4{\text{H}}_{2}\text{O}. \end{array}$$

For inorganic acids, temperature, pH value, reaction time, additives and so on have a significant impact on the leaching performance. Li et al. (2009) reported that under the condition of 80°C, 4M HCl solution was leached for 2 h, 99% Co and 97% Li were dissolved. Jha et al. (2013) reported that LiCoO_2_ was leaching 2M H_2_SO_4_ and 5% H_2_O_2_ (V/V) for 1 h at 75°C, achieving 99.1% Li dissolution and 70% Co dissolution. Chen et al. (2017) reported the precipitation of Co_3_(PO_4_)_2_ were separated from the solution containing lithium ions. And Shih et al. (2019) reported, in the presence of microwaves at 90°C, Co was completely dissolved and average 90% of the total metals were leached with H_2_SO_4_. What's more, more than 99.5% Mn was recycled from solution. Lee and Rhee (2003) reported that the solubility of Li and Co was over 95% at 75°C by leaching 1M HNO_3_ and 1.7% H_2_O_2_ (V/V) for 0.5 h (Pinna et al., 2016). In addition to strong inorganic acids, weak phosphoric acid has also proved to be a good solution for acidolysis. Pinna et al. (2016) reported the leaching performance at a concentration of 0.7M H_3_PO_4_ and 4% hydrogen peroxide (V/V), achieving a recovery of over 99% of Li and Co at 40°C for 1 h. The effect of ultrasonic and microwave on leaching reaction was studied (Shih et al., 2019).

In recent years, some mild organic acids have been widely studied. During oxalic acid leaching, leaching and precipitation usually occur simultaneously, resulting in CoC_2_O_4_ precipitation and the separation directly from the Li^+^ solution without further treatment. In addition, because oxalic acid solutions are reductive, no additional reductants are required. Liang and Qiu (2012) reported a recovery rate of more than 98% of Li^+^ and Co^2+^ in 1M acetic acid for 2 h at 80°C. Chen X. et al. (2016) reported the leaching performance using citric acid. The recovery of 99% Li, 93% Co, 91% Ni and 94% Mn was achieved by leaching the spent electrode material LiNi_1/3_Co_1/3_Mn_1/3_O_2_ at 80°C for 2 h. Chen et al. (2019) showed the recovery performance of 98% Co and 97% Li and transform spent LiCoO_2_ to precipitate and Li-enriched solution by tartaric acid.

As a hydrometallurgical process, bioleached metals are extracted by dissolving spent electrode materials with metabolites excreted by microorganisms (bacteria and fungi). Mishra et al. (2008) reported the performance of treating LiCoO_2_ by chemotrophic and acidophilic bacteria, and Niu et al. (2014) showed the leaching performance of Alicyclobacillus SP for Li and Co in different concentrations, that decreased, respectively, from 52 to 10% and from 80 to 37% with the increase of ore slurry concentration from 1 to 4%. Mohagheghi et al. (2017) reported the performance of Aspergillus Niger. The survival of fungi/bacteria was considered, so the bioleaching was affected by the slurry concentration. Bahaloo-Horeh and Mousavi (2017) reported the similar results of studies related to Aspergillus Niger and reported the effects of electrolytes in the slurry on biology.

### Pyrometallurgical Process

Pyrometallurgy is widely used for the commercial recovery of Co. The commonly used treatment of spent LIBs is similar to the ore smelting (Dunn et al., 2012). Before the smelting process, the modular LIBs are first disassembled into separate cells and then fed into a heating furnace. Batteries are reduced by preheating, pyrolysis and smelting, successively (Schakman et al., 2014; Jing et al., 2018). In the preheating zone, the heating temperature should be lower than 300°C to ensure complete evaporation of the electrolyte without explosion. And in the pyrolysis zone, the furnace temperature is controlled above 700°C. The purpose of this is to remove the plastic from the battery. In the smelting reduction zone, the material is smelted into alloys of Cu, Co, Ni, and Fe, along with Li, Al, Si, Ca, and some Fe slag. This method is usually only used to recover Cu, Co, Ni, and small amounts of Fe. Since Co plays an irreplaceable role in commercial LIBs, and thermal metallurgy has a high efficiency in recovering Co rather than Li, the economy of this recovery method depends largely on the amount of Co contained in spent LIBs and the fluctuation of market value of cobalt. However, as battery manufacturers consider the value and environmental issues of Co, Co-free electrode materials in LIBs have been continuously developed in recent years, significantly including LiMn_2_O_4_ and LiFePO_4_, some of which have been commercialized (Etacheri et al., 2011; Huang et al., 2014)^2^. Considering the limitation of lithium resources, this traditional method has no development prospect.

In order to recover Li from the spent LIBs, the selective pyrolysis method of an arc furnace can be used to convert some electrode materials into Co alloys and Li concentrate. After that, the Li is extracted by hydrometallurgy, and then it is transshipped and stored through the form of Li_2_CO_3_. And other components can be extracted further. The specific steps are shown in the Figure 2B. This method can be used not only to recycle electrode materials, but also to recycle Li and Fe, etc. in the electrolyte, which greatly improve the recovery efficiency.

Asadi Dalini et al. (2020) reported the usage scenarios and profit calculation of pyrometallurgy and hydrometallurgy used by different companies. Hydrometallurgy was mainly aimed at the recovery of Li_2_CO_3_ and Co_3_O_4_ and had 10,000-ton processing capacity. Anwani et al. (2020a) reported a laboratory-level life cycle assessment and economic analysis for cobalt oxalate from waste lithium ion batteries through acid leaching and roasting processes. For 3 g untreated positive electrode materials, the total process consumption of recovering cobalt oxalate was $0.59 and $0.67 for acid leaching and baking processes, respectively. Recently, Anwani et al. (2020b) reported the cost calculation of waste lithium ion batteries treated by acid leaching once again. According to the type of acid used in the process, the calculation for 10 g of untreated positive electrode material in 250 mL acid solution was carried out respectively. The cheapest oxalic acid was $1.06 and the most expensive acetic acid was $2.07.

Considering pyrometallurgy could cause the low recovery efficiency, high energy consumption and the production of toxic gases (dioxins, furans, etc.), the development of high recovery rate, low energy consumption and low environmental hazard recycling process has a good commercial prospect.

### Direct Physical Recycling Process

Direct recovery is a process of recovering useful components from spent LIBs without using chemical methods (Dunn et al., 2012; Yang et al., 2018). Before handling LIBs, they were discharged and disassembled into thousands of cells. Then, the small cells were treated with supercritical CO_2_, and the electrolytes were extracted and treated in this process. After lowering the temperature and pressure, CO_2_ can be separated from the electrolyte and the electrolyte can be regenerated. The cells were then disassembled, broken and sorted. Finally, the cathode material was collected and reused (Figure 2C).

Chen J. et al. (2016) reported a technology to directly recycle LiFePO_4_ from soft-pack batteries. In the case of no recovery of electrolyte, the spent LIBs were disassembled, crushed and cleaned in the sealed box. Due to residual polyvinylidene fluoride (PVDF) binder and material decomposition after thousands of charge and discharge cycles, the energy density of recovered LiFePO_4_ material is low and its electrochemical performance is poor. After heating treatment at 650°C, the electrochemical performance was improved, showing almost the same discharge capacity and energy density as the new material. Song et al. (2017) sintered the recovered materials with fresh powder through the physical direct recovery process to regenerate LiFePO_4_ materials from spent LIBs. The electrochemical performance of the regenerated LiFePO_4_ battery can meet the basic requirements of reuse. Huang et al. (2018) compared three direct physical recovery processes for graphite anode materials from spent LIBs. The first one was based on graphite heating treatment without recovery of the electrolyte. The second was to extract the electrolyte with subcritical CO_2_ and then heat it. The third was not only using supercritical CO_2_ as extraction agent, but also includes electrolyte extraction and heating treatment. Experimental results show that subcritical CO_2_ extraction electrolyte is the best recovery method. Zhang et al. (2019) reported the liberation efficiency of cathode materials was 98.23% by direct pyrolysis and physical recovery. Direct physical recovery technology has the advantages of short recovery route, low energy consumption, environmental friendliness and high recovery rate. However, it is not clear whether the recycled material will achieve the long-term properties of the new material.

## Conclusions and Prospects

In summary, all of the recycling processes discussed above are designed to recycle resources from spent LIBs. However, due to the different technical difficulties and economic benefits, the development stage of recycling process is not the same. Pyrometallurgy, for example, has been commercialized because of its simplicity and efficiency in recovering Co, the most valuable metal in the spent LIBs. With the development of battery technology, the content of Co in electrode materials is decreasing, while the use of Ni and manganese is increasing. In addition, the consumption of LIBs has grown rapidly in the past few years due to the rapid expansion of the EV market, and Li reserves have become a top concern. Therefore, the technology of Co recovery should be transferred to the comprehensive utilization of spent Li. In addition, when developing spent leaching recovery process, consideration should be given to appropriate treatment or recovery of certain materials that may pose a risk to the environment, such as electrolytes.

In addition, because the recovery of LIBs is in the initial stage, the current research level and the industrial development level cannot achieve the perfect recovery, namely safety, low cost, low energy consumption and no pollution. So further research and investment is inevitable. In order to better develop recycling technology of LIBs, it is best to do the following aspects: (1) Classify LIBs well according to the type of electrode; (2) Do a good job in battery design to make it meet the conditions of easy regeneration; (3) Cooperation on the recovery of spent LIBs and relevant legislative work around the world.

## Author Contributions

L-FZ organized and wrote the manuscript. HG and DY discussed the results. TD and W-BL guided the paper. All authors approved this manuscript.

## Conflict of Interest

The authors declare that the research was conducted in the absence of any commercial or financial relationships that could be construed as a potential conflict of interest.

## Footnotes

^1^https://www.zsw-bw.de/en/media-center/data-service.html#c8750

^2^http://www.chyxx.com/industry/201707/543308.html
